# Classification of pleural effusions using deep learning visual models: contrastive-loss

**DOI:** 10.1038/s41598-022-09550-w

**Published:** 2022-04-01

**Authors:** Jang Ho Lee, Chang-Min Choi, Namu Park, Hyung Jun Park

**Affiliations:** 1 Department of Pulmonary and Critical Care Medicine, Asan Medical Center, University of Ulsan College of Medicine, 88 Olympic-ro 43-gil, Songpa-gu, Seoul, 05505 South Korea; 2 Department of Oncology, Asan Medical Center, University of Ulsan College of Medicine, Seoul, South Korea; 3 Department of Biomedical Informatics and Medical Education, School of Medicine, University of Washington, Seattle, WA USA

**Keywords:** Computational biology and bioinformatics, Medical research

## Abstract

Blood and fluid analysis is extensively used for classifying the etiology of pleural effusion. However, most studies focused on determining the presence of a disease. This study classified pleural effusion etiology employing deep learning models by applying contrastive-loss. Patients with pleural effusion who underwent thoracentesis between 2009 and 2019 at the Asan Medical Center were analyzed. Five different models for categorizing the etiology of pleural effusion were compared. The performance metrics were top-1 accuracy, top-2 accuracy, and micro-and weighted-AUROC. UMAP and t-SNE were used to visualize the contrastive-loss model’s embedding space. Although the 5 models displayed similar performance in the validation set, the contrastive-loss model showed the highest accuracy in the extra-validation set. Additionally, the accuracy and micro-AUROC of the contrastive-loss model were 81.7% and 0.942 in the validation set, and 66.2% and 0.867 in the extra-validation set. Furthermore, the embedding space visualization in the contrastive-loss model exhibited typical and atypical effusion results by comparing the true and false positives of the rule-based criteria. Therefore, classifying the etiology of pleural effusion was achievable using the contrastive-loss model. Conclusively, visualization of the contrastive-loss model will provide clinicians with valuable insights for etiology diagnosis by differentiating between typical and atypical disease types.

## Introduction

The various etiologies for pleural effusion include infectious disease, malignant disease, volume overload, non-infectious inflammatory disease, and other systemic diseases^1^. Therefore, to avoid mistreatment or appropriate treatment delays that lead to respiratory and systemic complications, decreases in the quality of life, and even death, quick and accurate diagnosis of pleural effusion is crucial in clinical practice^2^. Diagnosis of pleural effusion depends on the judgment founded on pleural fluid cytology, biochemistry, clinical presentation, and the experience of clinicians. However, over 20% of the cases with etiology of pleural effusion remain unidentified despite various indicators^3,4^.

There have been several attempts to determine the etiology of pleural effusion using pleural fluid biomarkers^5^, ranging from transudates and exudates classification based on Light’s criteria^6^, tuberculous pleural effusion determination^7^, bacterial^8^, and complicated pleural effusion^9^. However, each biomarker was inefficient in defining the multiple etiologies of pleural effusion, resulting in some strategies adopting multinomial regression or decision tree to be considered^10,11^. Many studies have adopted the multi-class classification methods in deep learning, classifying multiple variables into three or more classes^12,13^. The cross-entropy loss is the most used loss function for multi-class classification. However, it has drawbacks such as the absence of robustness for noisy labels and poor discriminative margins possibilities^15^, suggesting easy bias in some models with cross-entropy loss function, especially those trained on small datasets.

Interestingly, to overcome this challenge, the method of contrastive-loss, which involves combining similar etiology’s inputs in the embedding space, separating those with different etiologies, has been developed^16^. Vanilla contrastive-loss learning is based on a triplet of which one pair is of a similar label, and the other differs^16^. Recently, Prannay et al. reported the development of supervised contrastive-loss learning that outperforms the cross-entropy loss for classifying annotated datasets. Also, since embedding space consists of high-dimensional space invisible in two-dimensional space, the visualization of high-dimensional space is important to allow readers to understand the effectiveness of the model method for classifying the etiology. However, most studies on contrastive-loss focused on analyzing image data instead of laboratory findings commonly used in the clinical field. Additionally, applying the contrastive-loss function and visualization of the embedding space enables the separation of different etiologies of pleural effusion informative embedding spaces.

Therefore, our study compared five different strategies, such as statistical method (multinomial logistic regression), machine learning-based models (random forest, gradient boost), and deep learning-based models (deep neural network, and contrastive-loss) to classify the etiology of pleural effusion using laboratory data and visualized the models’ embedding space by using two-dimensional reduction methods: Uniform Manifold Approximation and Projection (UMAP) and t-Stochastic Neighbor Embedding (t-SNE).

## Methods

### Clinical data

We retrospectively analyzed the medical records of patients who underwent cell analysis of pleural effusion between 2009 and 2019 at the Asan Medical Center (Seoul, South Korea). We randomly selected 1,918 patients for the training and validation datasets and finished training the model from the 3,799 patients. Among the 1,881 patients, 1,000 patients were randomly selected for the extra-validation dataset. The exclusion criteria were: (1) multiple causes as judged by two clinicians, (2) unclear etiology of the pleural effusion, or (3) pleural fluid cell analysis was only conducted for the follow-up after treatment, not for the initial diagnosis. Additionally, patient data were extracted from the in-house system (ABLE) and indexed by de-identified encrypted patient identification numbers to maintain confidentiality^17,18^. Laboratory data such as blood chemistry, complete blood cell count, pleural fluid cell count, and pleural fluid chemistry were analyzed and extracted according to the presence of pleural fluid cell count, mandatory in examining pleural tapping. These data were extracted at the acquisition date within two weeks of the pleural cell count. Also, the latest data were included for analysis when multiple laboratory results were obtained. Supplementary Table 5 describes the detailed information on the laboratory data.

The ethics committee of Asan Medical Center approved this study, conducted following the declaration of Helsinki. Also, the ethics committee of Asan Medical Center (approval number 2020–1157) waived the informed consent due to the retrospective observational nature of the study.

### Label classification and report annotation

We classified the etiology of pleural effusion into five categories to simplify the etiologies in our report. Considering the model prediction information for clinical practice, the model should recommend which practice to consider for each case. Accordingly, the label was classified as “bacterial infection,” “tuberculosis,” “malignancy,” “volume overload,” and “others.” Also, for the “others” category, the various non-infectious or non-bacterial conditions were included (Table 1). Furthermore, to compare the efficacy of models with previous rule-based definitions, the tuberculous and the complicated pleural effusion were diagnosed as follows: previous rule-based tuberculous effusion diagnosed when the pleural fluid fulfilled the following criteria: pleural adenosine deaminase (ADA) > 50 U/L and lymphocyte/neutrophil ratio > 0.75^1,7^; complicated pleural effusion diagnosed when the pleural fluid fulfilled two or more of these criteria: pH < 7.2, glucose < 60, lactate dehydrogenase (LD) > 1000^3,9^.

**Table 1:** The etiology distribution of pleural effusion in the datasets.

Etiology	Training and validation set (n = 1344)	Extra-validation set (n = 701)
Bacterial infection	201 (15.0%)	161 (23%)
Tuberculosis	123 (9.2%)	106 (15.1%)
Malignancy	859 (63.9%)	274 (39.1%)
Volume overload	95 (7.1%)	42 (6.0%)
**Others**		
Hemothorax	12 (0.9%)	14 (2.0%)
Hydropneumothorax	8 (0.6%)	10 (1.4%)
*Infection other than bacteria*	16 (1.2%)	14 (2.0%)
Parasite	4 (0.3%)	6 (0.9%)
NTM	3 (0.2%)	2 (0.3%)
Chronic empyema	3 (0.2%)	1 (0.1%)
Fungus	3 (0.2%)	0 (0.0%)
Viral	1 (0.1%)	3 (0.4%)
Nocardiosis	1 (0.1%)	0 (0.0%)
Scrub typhus	1 (0.1%)	0 (0.0%)
Actinomycosis	0 (0.0%)	2 (0.3%)
*Inflammation*	30 (2.2%)	80 (11.4%)
Autoimmune disease	4 (0.3%)	17 (2.4%)
PTE	4 (0.3%)	2 (0.3%)
Pleurodesis	3 (0.2%)	2 (0.3%)
Post-operation	2 (0.1%)	14 (2.0%)
Drug side effects	2 (0.1%)	13 (1.9%)
Paradoxical response TB	1 (0.1%)	7 (1.0%)
Pancreatitis	1 (0.1%)	0 (0.0%)
Trauma	0 (0.0%)	6 (0.9%)
Chylothorax	0 (0.0%)	1 (0.1%)
Other	13 (1.0%)	18 (2.6%)

Data are n (%).

NTM: nontuberculous mycobacteria; PTE: pulmonary thromboembolism; TB: tuberculosis.

We also labeled the etiology of pleural effusion for training and validation of models by following the described methods. Moreover, to facilitate disease annotation, the definite etiology of pleural effusion was auto-labeled as follows: cancer cells were identified in the pleural fluid cell analysis, *Mycobacterium tuberculosis* was cultured from the pleural fluid, or bacteria were cultured from the pleural fluid. We annotated them based on their clinical diagnosis due to the difficulty of labeling many patients as having definitive etiologies. The pleural fluid etiology annotation was based on chart reviews, independent of the pleural fluid results. Also, we defined a “clinically diagnosed tuberculosis pleural effusion” since less than half of the tuberculosis pleural effusions had a positive culture^19^. We defined it by the improvement of pleural effusion after 6 months with anti-tuberculosis treatment and one of the following conditions: (i) pleural effusion followed by a pulmonary tuberculosis lesion, (ii) pathologic findings of granuloma in the pleural biopsy, or (iii) tuberculosis suspected by imaging tests and tuberculosis drugs initiated according to the clinicians’ judgment. Here, we annotated the cause of the pleural effusion to be *Mycobacterium tuberculosis*. Conversely, we annotated that the pleural effusion caused by bacteria when using adequate antibiotics improved the symptoms and excluded other causes. Malignant effusion was defined when pleural metastasis was suspected in the imaging study, excluding other causes. Also, data from lost cases during follow-up treatment were excluded. Two clinicians reviewed the case and agreed on the label during a discrepancy. Alternatively, when two clinicians disagreed, we allocated these cases as “unknown etiology,” excluding them from the final dataset. Supplemental Table 6 shows the Cohen’s Kappa value of the two reviewers.

### Contrastive-loss model and visualization method

Multi-layer perceptron^20^ was used for the encoder in the multi-class classification, using contrastive-loss to employ cosine similarity to embed similar labels. The closeness of each embedding showed similar characteristics of the inputs, whereas a distant location revealed dissimilar inputs. Although the loss, the interpreted characteristics of inputs by the encoder was visualized. The two methods mapped the high-dimensional into two-dimensional spaces for visualization, including the UMAP^21^ and t-SNE^22^. Additionally, the visualized data were the raw laboratory data of pleural effusion and the embedding space of the contrastive-loss model in the training, validation, and extra-validation set. Readers could intuitively understand how the model performs and distinguish between usual and atypical laboratory findings by comparing the model’s conclusions with the raw laboratory data on the visualization map. Additionally, we compared the model’s performance with the statistical method (multinomial logistic regression), two machine learning-based methods (random forest, gradient boost), and another deep learning model with cross-entropy loss. The detailed model structure is described in the Supplement Methods, and the source code is uploaded in GitHub^23^.

### Statistical analysis

The accuracy of the model was evaluated with top-1 accuracy (highest probable prediction similar to the true label) and top-2 accuracy (one of the two highest probable predictions similar to the true label). Also, for the multi-class classification, we evaluated the model with the weighted-average area under the receiver operating characteristics (AUROC) and micro-AUROC^24^. The evaluation of one classification and the other based on prediction score was performed using AUROC to evaluate the model’s performance per label^25^. The model’s performance is compared with the findings of previous studies using AUROC calculated using one vs. the rest of the classifications^5,8,10–12^, where the target was a binary class. A confusion matrix of the top-1 prediction was also used to illustrate the distribution of the prediction and target labels. Statistical analysis was performed using Python 3.7.6.

## Results

### Data description

**Figure 1:**
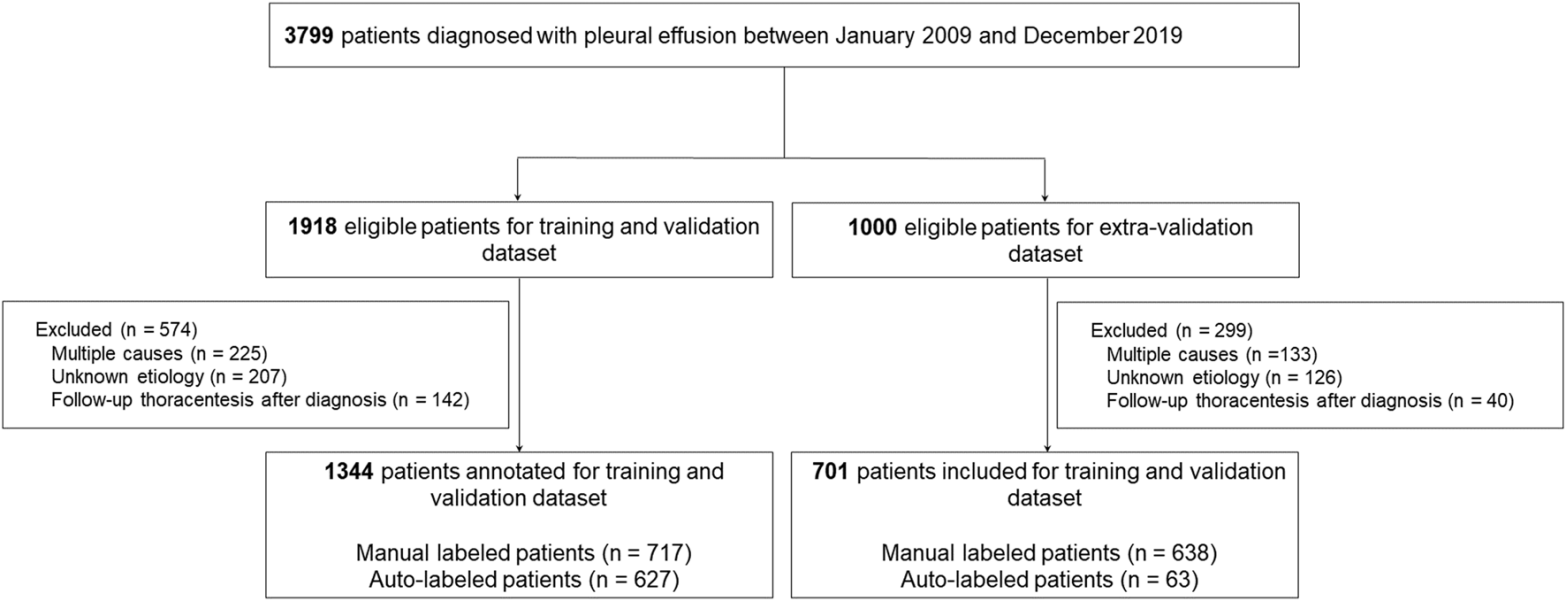
Patient flowchart.

Among the 3,799 cases of pleural effusion, 1,918 were manually and automatically annotated and used for the training and validation. Another 1,000 cases were manually annotated by two clinicians and used for the extra-validation. Among them, 574 patients in the training and validation dataset and 299 in the extra-validation dataset were excluded. Finally, 2,045 patients were included in this study, comprising 1,344 in the training and validation dataset and 701 in the extra-validation dataset (Fig. 1). According to the Light’s criteria, the exudate number was 3,358. Also, the number of complicated pleural effusions and confirmed malignant pleural effusions was 307 and 851, respectively. Table 1 describes the distribution of the etiology of pleural fluid according to the clinician’s annotation, and the detailed laboratory result of each label is described in Supplement Tables 1 and 2.

**Table 2:** Accuracy and AUROC of the applied models.

	Top-1 accuracy	Top-2 accuracy	Micro-AUROC	Weighted-AUROC
**Validation set**				
Multinomial logistic regression	80.5%	91.8%	0.961	0.941
Random forest	80.9%	92.1%	0.963	0.940
Gradient boost	82.9%	91.8%	0.966	0.947
Deep neural networks	79.4%	90.1%	0.935	0.904
Contrastive-loss model	81.7%	90.4%	0.942	0.913
**Extra-validation set**				
Multinomial logistic regression	60.6%	81.2%	0.853	0.827
Random forest	60.8%	80.9%	0.859	0.835
Gradient boost	62.9%	80.9%	0.860	0.838
Deep neural networks	65.2%	77.8%	0.843	0.821
Contrastive-loss model	66.2%	79.0%	0.867	0.819

AUROC: area under the receiver operating characteristic.

### Evaluation of the models in the validation set and the extra-validation set

We applied five different models for their performance in the validation set and the extra-validation set (Table 2): a statistical method (multinomial logistic regression), machine learning-based models (random forest, gradient boost), and deep learning-based models (deep neural network, and contrastive-loss). Results from the contrastive-loss model in the validation set revealed that the second-highest top-1 accuracy was 81.7%, with a rather low micro-AUROC at 0.942. Conversely, the contrastive-loss model showed the highest accuracy at 66.2% in the extra-validation set and the largest micro-AUROC at 0.867.

**Figure 2:**
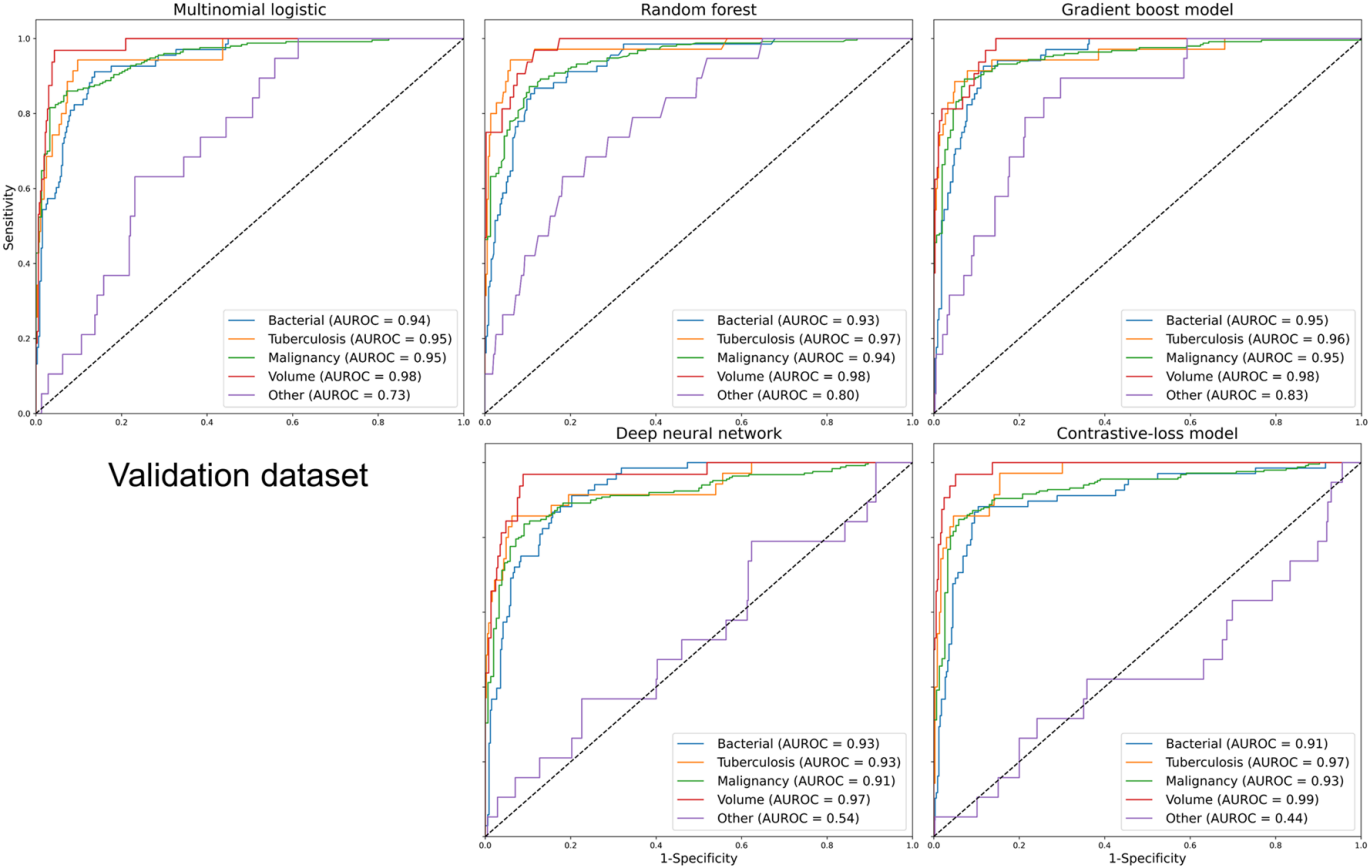
AUROC of each label in the validation dataset. AUROC: area under the receiver operating characteristic.

**Figure 3:**
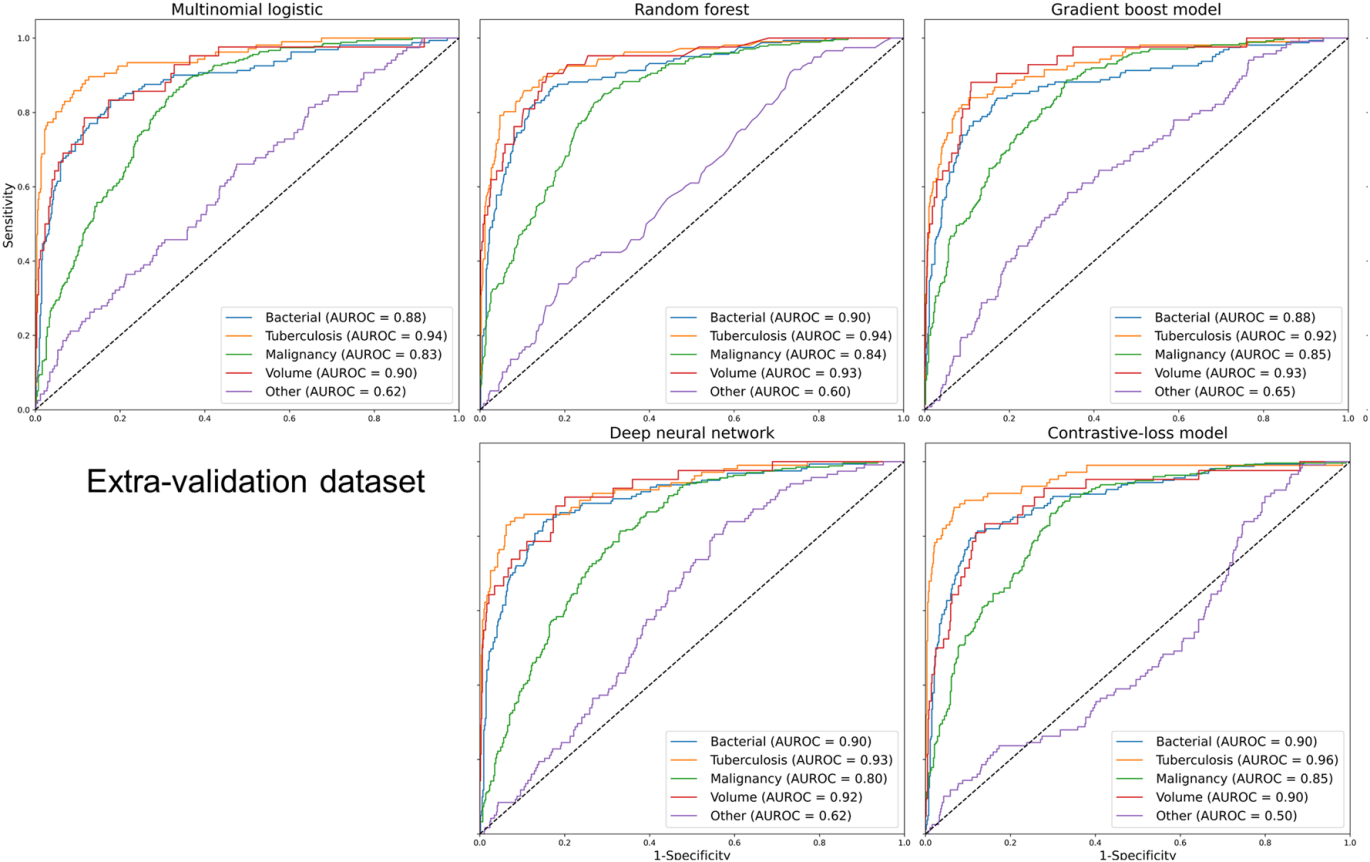
AUROC of each label in the extra-validation dataset. AUROC: area under the receiver operating characteristic.

Additionally, the AUROC for the categories of bacteria, tuberculosis, malignancy, and volume in terms of each etiology was above 0.9 in all validation set models (Fig. 2). However, the models showed a significant difference in the AUROC curves for the “other” category, indicating higher values in the gradient boost models (0.83) than in the contrastive-loss models (0.44). Alternatively, the AUROC of categories except for “other” in the extra-validation set was above 0.85 in all models. However, the AUROC curve of the “other” category was not preserved in the gradient boost model (0.65) (Fig. 3). Notably, the prediction of etiology showed a lower performance since the extra-validation set had a higher proportion of the “other” categories (Table 1). Also, when the confusion matrix revealed the prediction for each category, most categories were well-predicted except for the “other” category, which was predicted as malignancy (Supplement Table 3).

### Visualization of embedding space of the contrastive-loss model

**Figure 4:**
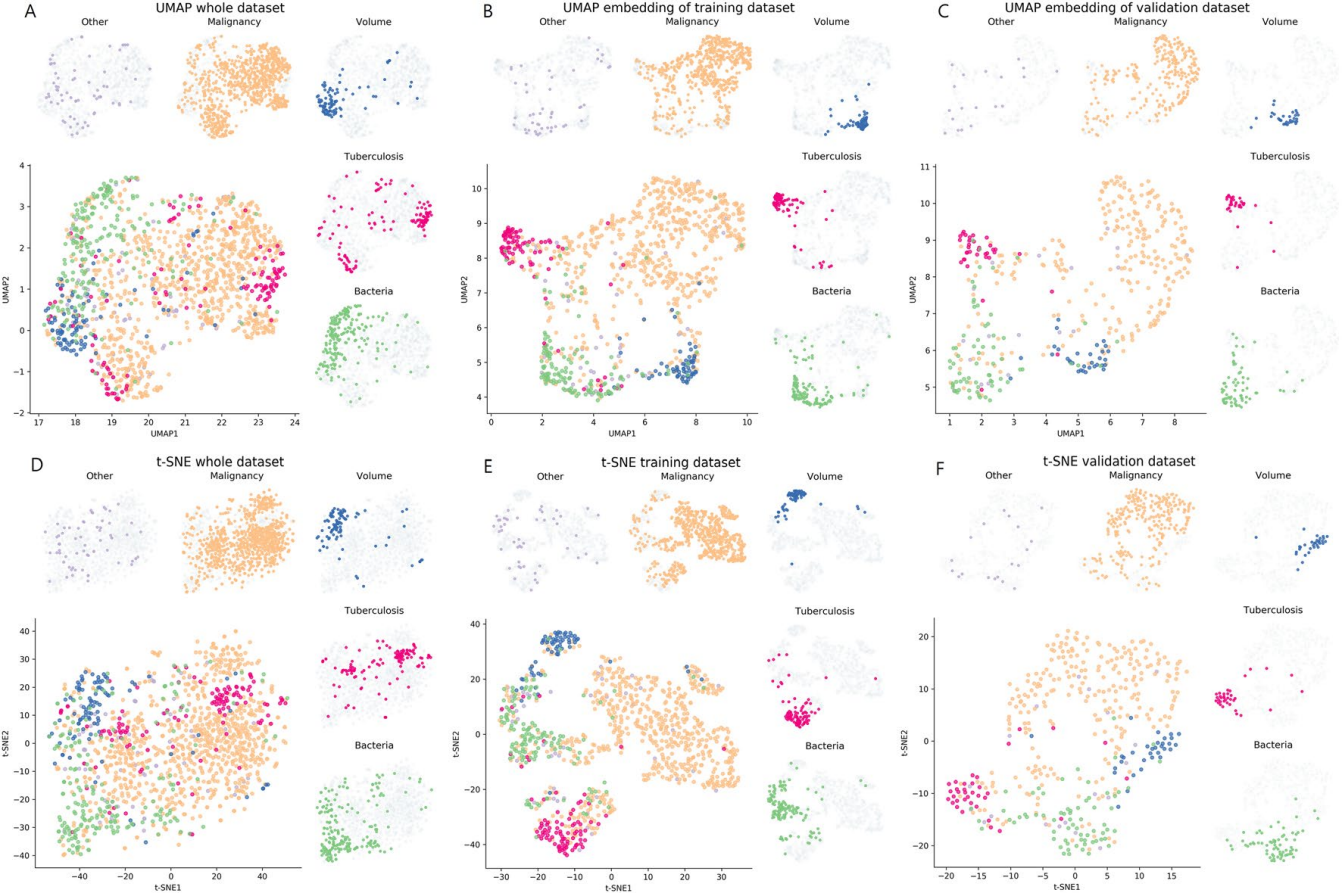
Embedding space of the contrastive-loss model in the validation dataset. The raw laboratory results are visualized in the embedding space of UMAP and t-SNE. (**A**, **D**) When the results are passed through the encoder of the contrastive-loss model, the laboratory results are divided according to each etiology in the training set (**B**, **E**) and validation set (**C**, **F**).

We used the UMAP and t-SNE methods to visualize high-dimensional data into 2-dimension space, which provides insight into the label’s distribution to readers before and after passing the encoders. The embedding space of the raw laboratory data revealed some local distributions according to each label before passing the encoder of the contrastive-loss model (Fig. 4A, D). The raw values of laboratory findings were located in different regions according to the etiology without passing the encoder. The tuberculosis was situated in two different regions, however, “others” were sited without different regions. Additionally, after passing the encoder, the embedding space displayed more clustered loci according to each etiology in the training set (Fig. 4B, E) and validation set (Fig. 4C, F). Therefore, since the contrastive-loss measures the nearness of laboratory results to each etiology, the central loci of each cluster illustrate the typical result of each etiology.

### Comparison with previous rule-based criteria for classifying etiology

**Figure 5:**
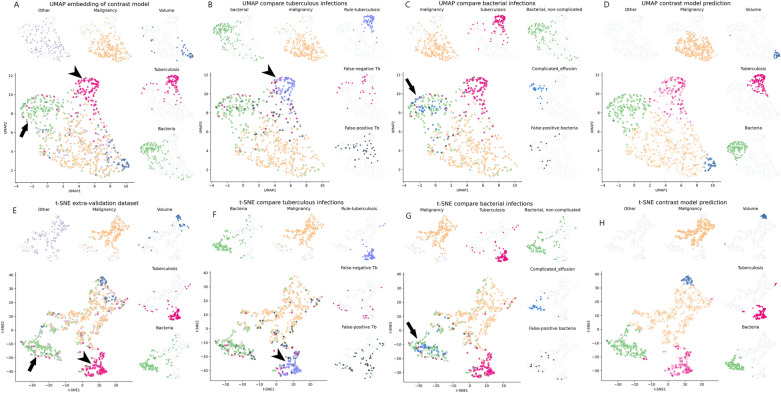
Comparison of the contrastive-loss model prediction with the rule-based label in the extra-validation set. Rule-tuberculosis: true-positive of the rule-based tuberculosis criteria; Rule-complicated effusion: true-positive of the rule-based complicated effusion. Arrowhead indicates the embedding area of tuberculosis in UMAP (**A, B**) and t-SNE (**E, F**), and many true-positive of rule-based tuberculosis is located in the tuberculosis area. The arrow indicates the embedding area of bacterial infection, and the blue (complicated effusion) is located at the center of the bacterial infection area. (**A, C, E, G**) The predicted probability of the contrastive-loss model is visualized according to each etiology, in which a higher density indicates higher prediction probability. (**D**, **H**).

Additionally, to understand the meaning of contrastive-loss embedding, true-positive and false-positive of the loci employing rule-based criteria were situated in the embedding map in the extra-validation set (Fig. 5). Also, Fig. 5B and F also depict the true-positive, false-positive, and false-negative loci using rule-based tuberculosis criteria. The true-positive loci were situated in the tuberculous region, whereas most false-positive tuberculosis was located in the bacterial or malignancy regions. The sensitivity and specificity of our model were 84.1% and 94.1%, respectively, superior to those of the ADA-based criteria (80.2% and 90.3%, respectively). Furthermore, when compared with the complicated effusion, the true-positive was labeled as “complicated effusion,” the rest of the bacterial cause, the non-complicated effusion, was labeled as “bacterial, non-complicated,” and the non-bacterial cause of complicated effusion was labeled as “False-positive bacterial” (Fig. 5C, G). Interestingly, most true-positive complicated effusion was in the center of bacterial regions as complicated effusion, an advanced stage of bacterial infection is typical of the etiology status.

Additionally, the probability of the contrastive-loss model for each category was visualized on UMAP, and the t-SNE map with the darker color shades signified a higher probability (Fig. 5D, H). Also, the higher prediction probabilities were more clustered in the t-SNE map than in the UMAP. The true-positive of rule-based tuberculosis and complicated effusion showed greater probabilities in each prediction, as shown in the darker color.

## Discussion

This study is the first to demonstrate the visualization of laboratory results in diagnosing the etiology of pleural effusion, to the best of our knowledge, potentially assisting clinicians in making intuitive judgments when determining pleural effusion causes. The five models in this study showed comparable performance in the validation set, with the contrastive-loss model possessing the highest accuracy in the extra-validation set. Moreover, the visualization of the embedding space of the contrastive-loss model using t-SNE and UMAP showed visible features of each pleural fluid etiology than the true-positives and false-positives of the rule-based criteria. Additionally, this study provided a valuable tool for classifying the etiology of pleural effusions with visualization of the embedding space, aiding clinicians’ evaluation of the effusion analysis closeness to each etiology group.

However, our contrastive-loss model produced embeddings easily viewed by UMAP and t-SNE. The embedding map was interpreted as an etiology likelihood map since the inputs with similar features were trained to have close loci in the embedding space^14^. A previous study showed that UMAP has a quicker computing speed than the t-SNE and best-preserved the global data structure^26^. Moreover, the UMAP reproduced the same embedding map at a fixed random seed of the model, whereas the t-SNE did not reproduce the same embedding map. The visualization method of the embedding space showed the probability of prediction and the aleatoric uncertainty of the data with an embedding map distance from the new data of each etiology region. Therefore, embedding maps helps clinicians decide the etiology of pleural effusion without understanding the difficult explanation of uncertainty in the Bayesian method.

Furthermore, previous studies showed advantageous indicators in diagnosing each cause of the pleural effusion^5^. However, a limited number of studies have classified the etiology of pleural effusion using the multi-class classification. Statistical models such as multinomial regression identify what variables affect the prediction in terms of odds ratio; however, the degree of odds ratios in the same direction with the multi-labels provide little information for classifying the etiology (Supplement Table 4). In actual clinical practice, it is difficult to differentiate between tuberculous and malignant pleural effusion, especially in the absence of additive findings for tuberculosis and malignancy. For example, though ADA is one of the best-studied indicators of tuberculosis, increased ADA levels are observed in parapneumonic effusion, empyema, malignant effusion, and rheumatoid arthritis-associated pleural effusion^27,28^. ADA could show a relatively low specificity, especially in such inflammatory conditions, confusing the endemic tuberculosis areas. Additionally, considering the diagnostic complexities, our model-assisted clinicians by providing quantified probability with visualization and the predicted etiology, revealing a better performance than in the previous ADA-based criteria^1,7^.

This method applies to clinical data with numerous laboratory results that lead to uncertainty in diagnosing the disease or determining the etiology. Moreover, as in our study result, the high performance of binary classification does not guarantee accuracy in multi-class classification. Conversely, the low accuracy of a model does not indicate that the classification model is impractical since some patients have definite features of some diseases while some do not. In this regard, our visualization method using the contrastive-loss model helps understand the favorable etiology, especially those confidently trusted and those that cannot. Therefore, this method helps acquire disease likelihood in situations with limited information.

There are several limitations to this study. First, all applied models’ accuracy was lower in the extra-validation set than in the validation set, attributed to the exclusion of definitive cases diagnosed using positive bacterial culture and malignant cells in the pleural fluid. Such exclusion requires the extra-validation dataset inclusion of cases with relatively obscure laboratory results than the validation set since patients characterized with auto-label are more definite than the negative culture effusion and exclusive diagnosis of malignant effusion. Another reason for the lower performance in the extra-validation set is the numerous cases in the “other” etiology category, without common features helpful for categorization. However, concerns exist about the overfitting issue. Therefore, we experimented with 100 times stratified splitting to investigate this point and presented the results in “Sensitivity analysis of the results.” Furthermore, we divided the datasets in this k-fold stratification analysis into training, validation, and test sets using stratification and validated our model trained on every divided dataset. The accuracy of the test set was not different from that of the validation set. Therefore, this result indicates that the down-performance in the extra-validation set of our main result was attributed to the different distributions of each dataset, not overfitting. Our validation sequence, model training, and testing in another annotated set were more reliable and represented a actual practical setting. Second, the probability of the model prediction unequaled the real probability that requires a calibration process. Also, when the model is applied in real-world practice, the probability calibration will be needed to avoid over-confidence and under-confidence. However, our model provides the embedding map to add credibility to the prediction, clinicians to discern typical findings that lead to the antibiotics or anti-tuberculosis drugs usage from the atypical findings, necessitating further evaluation to identify the etiology. Third, the “other” category was heterogeneous (Table 1), which could cause the low accuracy of the “other” category of our model. Additional studies with a large number of patients are required to improve the model in classifying the “other”. Finally, some ambiguous annotations exist due to the study’s retrospective nature, and since the cause of pleural effusion, the determination was not based on the gold standard. Therefore, researchers have attempted to determine annotation through discussion, excluding the disagreed cases to minimize this point.

Conclusively, our study demonstrates the usefulness of the contrastive-loss model in classifying the etiology of pleural effusion with visualization of the embedding space. We also expect clinicians to obtain insight into the closeness of the effusion analysis to each etiology by using this model, better diagnose the etiology of pleural effusion by differentiating typical and atypical disease types, including deciding the optimal initial treatment for pleural effusion.

## Supplementary Information

Supplementary Information.

## Data Availability

The data that support the findings of this study are available from the institutional review board of Asan Medical Center, while restrictions apply to the availability of these data that were used under license for the current study and so are not publicly available. However, data are available from the corresponding author upon reasonable request and with the permission of the institutional review board of Asan Medical Center.
