# Supplementary material for: Classification of pleural effusions using deep learning visual models: contrastive-loss
Source: Sci Rep. 2022 Apr 1;12:5532. doi: 10.1038/s41598-022-09550-w (PMC8975824; doi:10.1038/s41598-022-09550-w)
Supplement: Supplementary file 1 — Supplementary Information. [file 41598_2022_9550_MOESM1_ESM.docx]

**Supplement Methods and results**

**Visualization of the etiology of pleural effusion using contrastive-loss**

Jang Ho Lee^1^, Chang Min Choi^1,2^, Namu Park^3^, and Hyung Jun Park^1^,

**Table of contents**

**[Supplement Methods](#_Toc97936752)** [3](#_Toc97936752)

**[Applied models](#_Toc97936753)** [3](#_Toc97936753)

***[Multinomial logistic regression](#_Toc97936754)*** [3](#_Toc97936754)

***[Random forest](#_Toc97936755)*** [3](#_Toc97936755)

***[Gradient boost model](#_Toc97936756)*** [3](#_Toc97936756)

***[Deep feedforward networks](#_Toc97936757)*** [3](#_Toc97936757)

**[Preprocessing](#_Toc97936758)** [4](#_Toc97936758)

**[Encoder and projection network](#_Toc97936759)** [4](#_Toc97936759)

[Supplement Figure 1. The Structure of Encoder and Classifier network. 5](#_Toc97936760)

**[Contrastive-loss functions](#_Toc97936761)** [5](#_Toc97936761)

**[Training and hyperparameters](#_Toc97936762)** [6](#_Toc97936762)

**[Mini-batch sampling method](#_Toc97936763)** [6](#_Toc97936763)

[Number of hidden layers of the deep neural network and contrastive-loss model 7](#_Toc97936764)

**[Training and validation set description](#_Toc97936765)** [7](#_Toc97936765)

**[Sensitivity analysis of the results](#_Toc97936766)** [8](#_Toc97936766)

**[Supplement Table 1. Summary statistics of the laboratory results according to etiology in the training dataset.](#_Toc97936767)** [9](#_Toc97936767)

**[Supplement Table 2. Summary statistics of the laboratory results according to etiology in the extra-validation dataset.](#_Toc97936768)** [12](#_Toc97936768)

**[Supplement Table 3. Confusion matrix of the validation set and extra-validation set predicted by the contrastive-loss model](#_Toc97936769)** [15](#_Toc97936769)

**[Supplement Table 4. The odds ratio of each laboratory result by multinomial logistic regression](#_Toc97936770)** [16](#_Toc97936770)

**[Supplement Table 5. List of extracted chemical categories for blood and pleural effusion.](#_Toc97936771)** [18](#_Toc97936771)

**[Supplement Table 6. Interobserver agreement and Cohen’s Kappa in the extra-validation dataset.](#_Toc97936772)** [20](#_Toc97936772)

**[Supplement Table 7. List of 46 categories for model development.](#_Toc97936773)** [21](#_Toc97936773)

**[References](#_Toc97936774)** [22](#_Toc97936774)

# **Supplement Methods**

## **Applied models**

### ***Multinomial logistic regression***

For binary outcomes, logistic regression model is the most frequently used model. In case of multi-class outcomes, logistic regression model can be extended to multinomial logistic regression that does not have a natural ordering of the categories.^1^ Furthermore, because the characteristics of the dataset’s outcomes did not have a natural order, each outcome was compared to the reference outcome, “volume overload,” using the odds ratio of each dependent variable. In the package of multinomial logistic regression in scikit learn,^1^ newton-cg was used as the solver, and L2-regularization was used.

### ***Random forest***

The random forest model generates multiple decision trees using bagging and bootstrap aggregation of datasets. The random forest model predicts the outcomes with an ensemble of the decision trees’ predictions.^2^

### ***Gradient boost model***

Whereas the random forest model averages simple models, the gradient boosting model adds new models to the ensemble in sequence. Adding new weak models reduces the loss function, and the ensemble of the models thus has better prediction.^2^

### ***Deep feedforward networks***

The deep feedforward network consisted of three or four hidden layers and an output layer by receiving each row of the laboratory data as an input.^3^ Each node of the hidden layer consisted of 64 to 1024 nodes. Except for the last layer, which was added with the sigmoid function as the activation function, every hidden layer is added with the Relu function as the activation function^4^ Loss function of the model was a cross-entropy loss. Detailed hyperparameters were the same as the contrastive-loss model.

## **Preprocessing**

We interpolated the data with its “mode” for omitted data, which can signify the most non-informative value. The majority of the data was skewed and tended to have a long tail to an extremely high level of each lab. We assumed that the most common number indicates the average value and that it might be used to replace missing data. As a result, we used the “mode” value to interpolate the data. The model would be difficult to train if too much data was excluded; therefore, we selected lab data in which no more than 60% of the total data was omitted. (Supplement Table 7)

The laboratory data were capped at the 5^th^ and 95^th^ percentiles to rule out outliers. Then, using the MinMaxScaler,^5^ the range of the data was scaled from 0.01 to 1. Although most of the data were right-skewed, we did not transform the distribution because the transformation of data distribution does not significantly help the model perform better, and transformation is not easy to generalize in other datasets.

##

## **Encoder and projection network**

Encoder network, $Enc\left( \boldsymbol{x} \right),$maps *x* to a representation vector, $\mathcal{Z} = Enc(\boldsymbol{x}) \in R^{D_{E}}$. $\boldsymbol{r}$ is normalized to the unit hypersphere in $R^{D_{E}}$ ($D_{E}$ = 20 in our investigation). A multi-layer perceptron served as the encoder layer^3^ with an activation function of sigmoid and Relu.^4^ Every layer was normalized by Batch-Normalization.^6^ The dimension of $\boldsymbol{x}$ was 40, which is the number of pleural effusion biomarkers (Supplement Table 7). The dimension of $\mathcal{Z}$ was less than that in an earlier article^7^ ($D_{E}$ = 2048) due to the less complexity of our pleural effusion data.

Classifier network, $Classifier(\mathcal{Z})$ comprised a linear classifier. The output vector of size $D_{p}$ was 5, which is the number of pleural fluid etiologies (Supplement Figure 1).

## Supplement Figure 1. The Structure of Encoder and Classifier network.


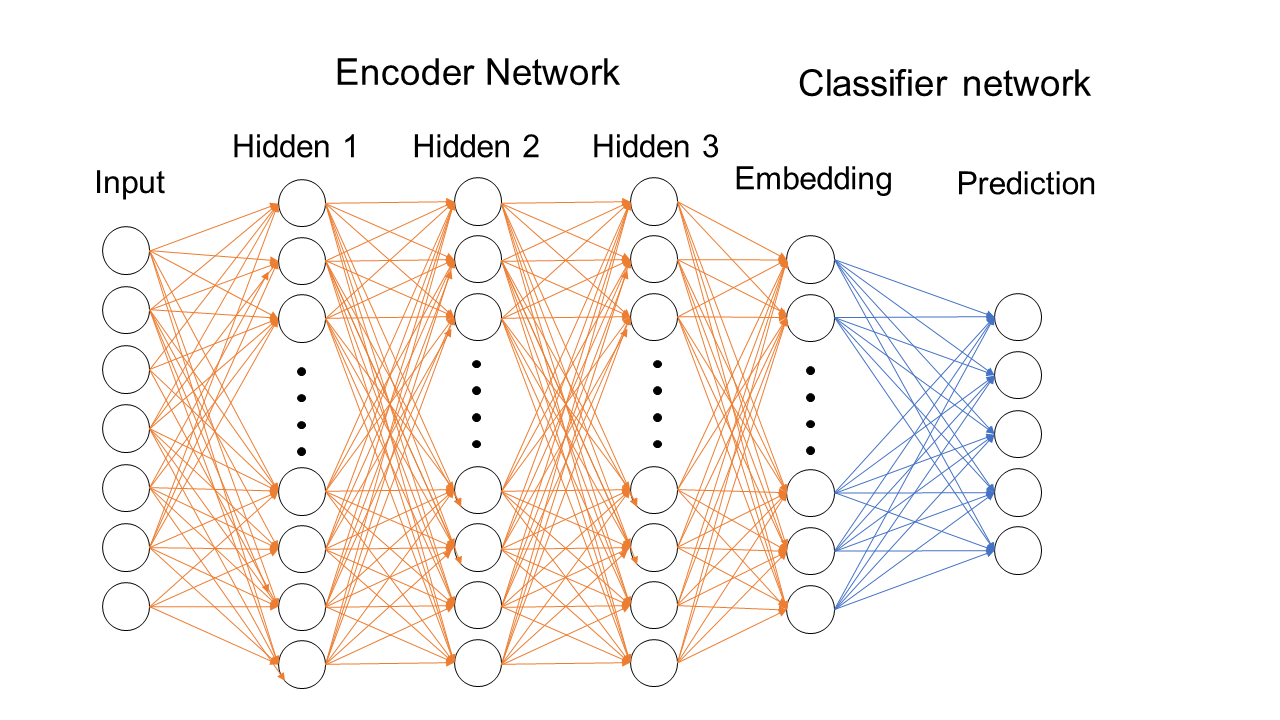


## **Contrastive-loss functions**

We adopted the self-supervised contrastive-loss^7^, which uses the positive sample as augmented and the same class data. A batch is randomly picked to sample/label pairings for a set of N, {x_k_, y_k_}_k=1.N_. The number of y classes was five since the etiology of pleural effusion was divided into five types. Each laboratory data of pleural effusion were represented by X_k_.

Furthermore, because the last layer of the encoder network represented each input as an embedding, the similarity of the embeddings was conveyed by normalizing the embeddings and substituting the Euclidean distance with inner products.^7^ Let $i\in I \equiv\{1...N\}$ be the index of sample, and $z_{\iota} = Enc(\boldsymbol{x}_{\boldsymbol{\iota}}) \in R^{D_{E}}$, and the distance of $i$ (anchor) with positive sample is $z_{i}\cdot z_{\mathcal{p}}$. ($z_{\mathcal{p}}$is an embedding of positive sample). The distance addition of $i$ (anchor) with the rest of all the inputs is $\sum_{a\in A(i)} exp(z_{i}\cdot z_{a})$, where $A(i) \equiv I\backslash\{i\}$.

Positive samples should have closer positions in embedding encoder spaces, whereas negative samples should have farther positions. The following is an example of a loss in this context:

$Loss=\sum_{i\in I} {Loss}_{i}= \sum_{i\in I} \frac{-1}{\mathcal{|P(}i)|}\sum_{\mathcal{p\in P(}i)} log\frac{exp(z_{i}\cdot z_{\mathcal{p}}/\tau)}{\sum_{a\in A(i)} exp(z_{i}\cdot z_{a}/\tau)}$ (1)

, where $P(i) \equiv\{p \in A(i) : Y_{p} = Y_{i}\}$ is the set of all positives in the multi-viewed batch distinct from $i$, and $|P(i)|$is its cardinality.

## **Training and hyperparameters**

All variables were initialized using the normalized initialization method for training the deep neural network model and the contrastive-loss model (Xavier),^8^ the default initializer of PyTorch,^9^ and trained using the Adam Optimization^10^ with a learning rate of 2e-3 and weight decay of 1e-5. Based on the loss function, we optimized the hyperparameter with early stopping during the training. However, the minimal training epoch was set to 600 to avoid too early stopping due to inappropriate initializing. The detailed method of hyperparameter tuning is described in (<https://github.com/podkd87/Pleural-effusion/blob/main/contrastiveloss%20model%20training.ipynb>).

The grid-search technique was used to optimize the random forest and the gradient boost model for hyperparameter optimization. The maximum features in the random forest model ranged from 0.1 to 1.0, and the estimator number was 300, 1000, 3000, or 30000. The learning rate was searched with 0.3, 0.03, 0.003, or 0.0003 in the gradient boost model, while the number of estimators was searched with 300, 1000, 3000, or 30000.

## **Mini-batch sampling method**

The fraction of labels would be labels when each batch randomly selects examples from the training set. We used the Torch packages’ Weighed Random Sampler to train the model with the same weight among labels. The sampler could get each batch with the same number of each label by the weight that the number of total cases used as the denominator. (1/N)

## Number of hidden layers of the deep neural network and contrastive-loss model

We used various hidden layers for training the deep learning and contrastive-loss model. Both models used the same hidden layers to compare the effect of supervised contrastive loss with the cross-entropy loss. Below are the hidden layers used to train the model. Input layer = 46, output layer = 20, 5 (contrastive loss model, deep learning model)

| Contrastive loss model | Deep learning model |
| --- | --- |
| [128, 256, 128, 32] | [128, 256, 128, 32, 20] |
| [256, 512, 128, 64] | [256, 512, 128, 64, 20] |
| [256, 256, 256, 256] | [256, 256, 256, 256, 20] |
| [512, 512, 512, 512] | [512, 512, 512, 512, 20] |
| [1024, 1024, 1024] | [1024, 1024, 1024, 20]] |

After training the contrastive loss model with 20 dimension space, another classifier with [128,5] hidden layer space was used.

# **Training and validation set description**

Following excluding instances with various causes (n = 225), uncertain etiology after chart review (n = 207), and thoracentesis as a result of follow-up operations after treatment (n = 142) from the dataset of 1918 cases, 1344 cases remained. We divided the dataset into two parts: training (70%) and validation set (30%). The training and validation sets were split without stratified label splition using the Python Sklearn module. The label proportions vary slightly across the training and validation sets, as seen in the table below.

|  | Training Set (Number, %) | Validation Set (Number, %) |
| --- | --- | --- |
| Malignancy | 609 (64.79%) | 250 (61.88%) |
| Bacterial infection | 133 (14.15%) | 68 (16.83%) |
| Tuberculosis | 88 (9.36%) | 35 (8.66%) |
| Volume overload | 63 (6.7%) | 32 (7.92%) |
| Others | 47 (5%) | 19 (4.7%) |

# **Sensitivity analysis of the results**

The extra-validation set had a different class distribution than the training and validation sets since definitive cases (bacterial culture positive and malignant cell in pleural fluid) were used in training and validation set. As a result, the extra-validation set’s model accuracy was lower than the training and validation set. If the dataset were split randomly into training, validation, and test sets, the model performance would differ between the validation and test sets. Therefore, we experimented with 100 times stratified splitting to investigate this point. The following results support our previous conclusions.

The contrastive loss model was trained using each split set by employing hyperparameters from our previous best model, such as the total and hidden layer numbers, learning rate (0.0005), and weight decay (0.0001). We used stratification to divide the datasets into training, validation, and test sets 100 times and verified our model trained on each split dataset in this k-fold stratification analysis. We trained our model after splitting, and the best model based on the validation set was chosen to validate the performance in the test set. A hundred training sessions were completed, with each data split being random.

|  | Micro AUROC | Weighted AUROC | Top-1 Accuracy | Top-2 Accuracy |
| --- | --- | --- | --- | --- |
| Validation set | 0.93 (0.01) | 0.89 (0.01) | 75.13 (1.87) | 88.47 (1.38) |
| Test set | 0.93 (0.01) | 0.89 (0.01) | 75.1 (1.49) | 88.47 (1.16) |

* mean, (standard deviation)

The training code can be obtained from (github.com/podkd/plerual-effusion/contrastiveloss_revised2201251.ipynb)

This indicates that the underperformance in the extra-validation set of our main result was due to the different distributions of the dataset classes rather than overfitting. Despite the fact that we may use this outcome, our training, validation, and extra-validation scenario illustrates the genuine model validation procedure. Despite our model’s poor performance in the extra-validation set, we believe that our visualization could be useful for interpreting laboratory results in terms of pleural effusion causation. Furthermore, we believe that our validation procedure of training the model and testing it in another annotated set is more reliable and realistic.

# **Supplement Table 1. Summary statistics of the laboratory results according to etiology in the training dataset.**

|  | Bacterial infection | Tuberculosis | Malignancy | Volume overload | Others |
| --- | --- | --- | --- | --- | --- |
| Age | 69.49 ± (12.75) | 62.63 ± (18.97) | 68.17 ± (11.21) | 75.58 ± (9.26) | 67.03 ± (12.32) |
| Height (cm) | 161.28 ± (15.1) | 162.54 ± (15.15) | 160.99 ± (12.97) | 160.49 ± (8.84) | 163.43 ± (8.62) |
| Weight (kg) | 61.45 ± (11.98) | 61.28 ± (15.78) | 60.2 ± (12.29) | 58.83 ± (10.2) | 57.97 ± (9.29) |
| Histiocyte (pleural) | 12.33 ± (13.32) | 18.98 ± (13.94) | 27.73 ± (19.36) | 43.71 ± (22.64) | 20.26 ± (17.18) |
| Neutrophil (pleural) | 57.92 ± (36.32) | 16.55 ± (22.45) | 15.66 ± (23.81) | 16.64 ± (23.11) | 38.73 ± (32.63) |
| RBC (pleural) | 64757.83 ± (305598.26) | 71053.01 ± (424628.49) | 70573.56 ± (280354.24) | 52571.98 ± (336862.04) | 186981.06 ± (501270.59) |
| Eosinophil (pleural) | 1.75 ± (2.57) | 2.58 ± (5.32) | 2.81 ± (5.25) | 2.59 ± (6.72) | 12.44 ± (22.4) |
| Nucleated cells (pleural) | 39142.88 ± (94162.41) | 6243.78 ± (20661.36) | 7333.64 ± (47885.86) | 1186.41 ± (2684.34) | 7868.76 ± (19886.84) |
| Lymphocyte (pleural) | 16.55 ± (22.47) | 61.78 ± (25.62) | 35.14 ± (24.62) | 33.88 ± (22.01) | 28.45 ± (27.2) |
| Mesothelial cell (pleural) | 2.39 ± (4.38) | 1.83 ± (1.74) | 2.58 ± (3.81) | 3.41 ± (4.58) | 2.86 ± (3.55) |
| ADA (pleural) | 102.6 ± (140.7) | 87.27 ± (47.96) | 29.91 ± (57.37) | 10.65 ± (7.43) | 34.36 ± (30.56) |
| LD (pleural) | 5129.02 ± (10607.59) | 649.02 ± (1490.62) | 1245.14 ± (5934.22) | 127.06 ± (86.9) | 836.18 ± (1307.34) |
| Albumin (pleural) | 1.98 ± (0.76) | 2.67 ± (0.61) | 2.67 ± (0.73) | 1.43 ± (0.6) | 2.15 ± (0.78) |
| pH (pleural) | 6.97 ± (0.39) | 7.06 ± (0.16) | 7.11 ± (0.2) | 7.1 ± (0.18) | 7.01 ± (0.3) |
| Total protein (pleural) | 4.02 ± (1.37) | 4.82 ± (1.15) | 4.49 ± (1.07) | 2.45 ± (1.06) | 3.95 ± (1.16) |
| Glucose (pleural) | 81.45 ± (77.15) | 103.31 ± (50.08) | 105.26 ± (55.98) | 161.41 ± (58.66) | 107.42 ± (59.43) |
| Total amylase (pleural) | 30.89 ± (32.98) | 34.68 ± (18.67) | 89.67 ± (659.54) | 27.19 ± (28.19) | 27.53 ± (13.16) |
| Creatinine | 1.19 ± (1.28) | 0.86 ± (0.4) | 0.91 ± (0.87) | 1.43 ± (1.32) | 1.4 ± (2.1) |
| AST (SGOT) | 33.7 ± (27.54) | 29.36 ± (26.86) | 28.11 ± (37.72) | 47.79 ± (67.22) | 33.32 ± (44.75) |
| Alkaline phosphatase | 117.22 ± (75.71) | 90.21 ± (55.04) | 102.21 ± (94.89) | 99.27 ± (73.14) | 133.27 ± (357.56) |
| Total bilirubin | 1.06 ± (1.59) | 0.61 ± (0.29) | 0.56 ± (0.35) | 1.12 ± (1.65) | 0.6 ± (0.31) |
| Glucose | 147.51 ± (66.06) | 122.65 ± (49.9) | 133.58 ± (54.91) | 139.68 ± (56.69) | 139.61 ± (56.89) |
| Albumin | 2.58 ± (0.63) | 3.15 ± (0.58) | 3.17 ± (0.6) | 2.92 ± (0.61) | 2.86 ± (0.78) |
| BUN | 19.68 ± (13.33) | 14.33 ± (9.2) | 15.96 ± (9.9) | 22.29 ± (13.63) | 17.74 ± (8.54) |
| Total protein | 6.29 ± (0.94) | 6.68 ± (0.82) | 6.5 ± (0.7) | 6.23 ± (0.93) | 6.35 ± (0.94) |
| LD | 236.39 ± (123.13) | 216.01 ± (69.71) | 298.02 ± (372.56) | 284.23 ± (115.97) | 238.44 ± (99.5) |
| ALT (SGPT) | 27.94 ± (28.97) | 24.4 ± (32.39) | 23.18 ± (40.5) | 42.73 ± (96.01) | 31.89 ± (64.74) |
| r-GT | 59.75 ± (134.13) | 28.41 ± (36.79) | 33.68 ± (64.03) | 37.8 ± (42.5) | 29.02 ± (29.4) |
| CRP | 14.48 ± (9.18) | 5.64 ± (6.4) | 3.51 ± (5.54) | 3.16 ± (4.9) | 5.69 ± (6.89) |
| D-dimer | 16.92 ± (16.27) | 24.84 ± (15.03) | 25.97 ± (14.45) | 16.22 ± (15.92) | 17.31 ± (16.51) |
| BNP | 214.07 ± (446.65) | 77.96 ± (177.88) | 52.84 ± (198.93) | 811.26 ± (1263.6) | 248.89 ± (850.81) |
| Procalcitonin | 2.96 ± (10.23) | 0.14 ± (0.41) | 0.29 ± (3.11) | 0.57 ± (2.26) | 0.17 ± (0.3) |
| Total calcium | 8.35 ± (0.59) | 8.67 ± (0.71) | 8.94 ± (0.69) | 8.42 ± (0.68) | 8.5 ± (0.69) |
| Sodium | 135.91 ± (4.48) | 137.48 ± (3.51) | 138.09 ± (4.2) | 136.52 ± (5.29) | 137.17 ± (4.09) |
| Chloride | 99.83 ± (5.24) | 101.4 ± (3.75) | 101.02 ± (4.39) | 100.51 ± (7.07) | 100.65 ± (4.13) |
| Potassium | 4.15 ± (0.61) | 4.23 ± (0.39) | 4.3 ± (0.51) | 4.2 ± (0.59) | 4.27 ± (0.5) |
| Phosphorus | 3.1 ± (0.8) | 3.25 ± (0.6) | 3.5 ± (0.67) | 3.49 ± (0.87) | 3.48 ± (1.06) |
| Hb | 11.21 ± (2.08) | 12.52 ± (1.95) | 12.69 ± (1.87) | 11.11 ± (2.27) | 12.14 ± (2.22) |
| RDW | 14.3 ± (2.02) | 13.65 ± (1.74) | 13.5 ± (1.49) | 15.71 ± (2.31) | 14.2 ± (2.02) |
| WBC | 12.9 ± (7.08) | 6.99 ± (2.43) | 8.89 ± (4.07) | 8.52 ± (4.97) | 8.13 ± (4.58) |
| MCHC | 33 ± (1.4) | 33.17 ± (1.24) | 33.22 ± (1.1) | 32.88 ± (1.24) | 33.07 ± (1.15) |
| MCV | 91.7 ± (6.91) | 90.49 ± (6) | 91.83 ± (5.26) | 93.59 ± (6.54) | 91.12 ± (4.84) |
| PDW | 10.81 ± (3.11) | 10.84 ± (2.53) | 10.62 ± (1.95) | 11.94 ± (3.21) | 11.08 ± (1.68) |
| Hct | 33.9 ± (5.85) | 37.72 ± (5.25) | 38.21 ± (5.36) | 33.76 ± (6.71) | 36.6 ± (6.27) |
| E-ANC | 10082.29 ± (6054.57) | 4648.46 ± (1928.35) | 6282.01 ± (3850.09) | 6309.47 ± (4816.98) | 5825.91 ± (4230.82) |
| MCH | 30.28 ± (2.72) | 30.04 ± (2.32) | 30.51 ± (2) | 30.78 ± (2.48) | 30.15 ± (2.03) |
| RBC | 3.72 ± (0.71) | 4.17 ± (0.65) | 4.16 ± (0.63) | 3.62 ± (0.74) | 4.02 ± (0.67) |
| MPV | 9.75 ± (1.9) | 9.67 ± (1.37) | 9.63 ± (1.25) | 10.28 ± (2.12) | 10.02 ± (0.85) |
| Platelet | 278.5 ± (131.17) | 296.78 ± (94.01) | 289.9 ± (104.13) | 200.21 ± (105.57) | 238.12 ± (90.79) |

ADA: Adenosine De-Aminase; ALT(SGPT): Alanine Transaminase; ANC: Absolute Neutrophil Count; AST(SGOT): Aspartate Transaminase; BNP: Brain Natriuretic Peptide; BUN: Blood Urea Nitrogen; CRP: C-Reactive Protein; Hb: Hemoglobin; LD: Lactate Dehydrogenase; MCH: Mean Corpuscular Hemoglobin; MCHC: Mean Cell Hemoglobin Concentration; MCV: Mean Corpuscular Volume; MPV: Mean Platelet Volume; PDW: Platelet Distribution Width; RBC: Red Blood Cell; RDW: Red cell Distribution Width; r-GT: Gamma-Glutamyl Transferase; WBC: White Blood Cell

# **Supplement Table 2. Summary statistics of the laboratory results according to etiology in the extra-validation dataset.**

|  | Bacterial infection | Tuberculosis | Malignancy | Volume overload | Others |
| --- | --- | --- | --- | --- | --- |
| Age | 62.25 ± (15.28) | 55.22 ± (17.09) | 64.92 ± (13.06) | 67.31 ± (12.04) | 60.16 ± (15.45) |
| Height (cm) | 162.27 ± (14.32) | 163.48 ± (16.26) | 163.08 ± (8.38) | 158.9 ± (16.94) | 163.97 ± (12.39) |
| Weight (kg) | 61.74 ± (15.04) | 61.7 ± (13.54) | 59.91 ± (10.66) | 55.4 ± (13.8) | 61.15 ± (11.41) |
| Histiocyte (pleural) | 13.94 ± (16.75) | 17.03 ± (14.9) | 26.49 ± (20.17) | 43.63 ± (19.39) | 28.61 ± (23.13) |
| Neutrophil (pleural) | 68.28 ± (32.85) | 17.64 ± (24.51) | 18.36 ± (22.62) | 9.21 ± (13.07) | 23.63 ± (26.83) |
| RBC (pleural) | 53466.91 ± (170550.64) | 20694.83 ± (124057.34) | 72287.99 ± (239844.47) | 11776.26 ± (45880.45) | 288919.62 ± (780588.52) |
| Eosinophil (pleural) | 3.69 ± (6.99) | 5.83 ± (11.1) | 6.2 ± (9.9) | 11 ± (21.36) | 16.13 ± (24.17) |
| Nucleated cells (pleural) | 33036.17 ± (99335.3) | 7293.92 ± (24357.62) | 2666.82 ± (5147.83) | 2979.4 ± (12975.69) | 5633.79 ± (14858.49) |
| Lymphocyte (pleural) | 17.33 ± (21.16) | 64.98 ± (26.47) | 48.74 ± (26.97) | 43.63 ± (19.1) | 37.16 ± (28.78) |
| Mesothelial cell (pleural) | 3.44 ± (3.93) | 3.23 ± (4.12) | 4.02 ± (4.37) | 3.28 ± (3.41) | 4.18 ± (5.57) |
| ADA (pleural) | 85.73 ± (146.12) | 90.64 ± (60.98) | 23.15 ± (13.64) | 26.69 ± (74.09) | 34.85 ± (34.59) |
| LD (pleural) | 4772.32 ± (10422.86) | 821.12 ± (2228.62) | 546.18 ± (768.79) | 660.24 ± (3028.17) | 835.74 ± (1614.1) |
| Albumin (pleural) | 2.08 ± (0.8) | 2.7 ± (0.6) | 2.44 ± (0.61) | 1.47 ± (0.67) | 2.43 ± (0.66) |
| pH (pleural) | 6.99 ± (0.36) | 7.04 ± (0.17) | 7.11 ± (0.23) | 7.05 ± (0.2) | 7.09 ± (0.22) |
| Total protein (pleural) | 4.32 ± (1.26) | 5.04 ± (0.82) | 4.22 ± (0.89) | 2.73 ± (1.14) | 4.48 ± (1.21) |
| Glucose (pleural) | 86.81 ± (83.46) | 97.78 ± (48.17) | 118.08 ± (54.61) | 143.59 ± (74.94) | 101.51 ± (56.27) |
| Total amylase (pleural) | 30.97 ± (46.64) | 74.76 ± (152.58) | 85 ± (246.01) | 28.43 ± (18.88) | 531.66 ± (3428.47) |
| Creatinine | 1.14 ± (1.69) | 0.86 ± (0.55) | 0.86 ± (0.71) | 1.82 ± (2) | 0.89 ± (0.51) |
| AST (SGOT) | 29.45 ± (22.38) | 25.81 ± (15.83) | 31.42 ± (78.99) | 36.31 ± (23.34) | 27.62 ± (33.78) |
| Alkaline phosphatase | 119.89 ± (75.09) | 87.93 ± (42.2) | 127.22 ± (274.77) | 108.62 ± (52.46) | 94.56 ± (60.37) |
| Total bilirubin | 0.68 ± (0.46) | 0.6 ± (0.39) | 0.55 ± (0.36) | 0.86 ± (0.73) | 0.61 ± (0.65) |
| Glucose | 140.16 ± (54.28) | 130.06 ± (66.1) | 129.7 ± (52.27) | 144.1 ± (96.13) | 120.02 ± (37.14) |
| Albumin | 2.62 ± (0.59) | 3.18 ± (0.5) | 3.02 ± (0.55) | 2.89 ± (0.73) | 3.18 ± (0.66) |
| BUN | 16.57 ± (12.11) | 12.91 ± (6.23) | 14.44 ± (6.45) | 28.89 ± (31.24) | 15.61 ± (11.37) |
| Total protein | 6.39 ± (0.93) | 7.03 ± (0.71) | 6.45 ± (0.72) | 6.56 ± (1.04) | 6.55 ± (0.86) |
| LD | 206.44 ± (61.96) | 220.89 ± (84.11) | 276.06 ± (192.41) | 248.47 ± (71.61) | 230.12 ± (117.33) |
| ALT (SGPT) | 29.16 ± (28.18) | 25.32 ± (30.28) | 22.53 ± (26.41) | 21.26 ± (17.03) | 23.25 ± (35.55) |
| r-GT | 85.52 ± (96.97) | 38.5 ± (44.65) | 58.06 ± (121.63) | 42.91 ± (38.5) | 40.26 ± (47.91) |
| CRP | 14.59 ± (10.55) | 5.58 ± (5.54) | 4.9 ± (6.11) | 2.54 ± (5.08) | 3.99 ± (6.1) |
| D-dimer | 4.04 ± (5.23) | 5.05 ± (5.9) | 3.95 ± (5.02) | 2.83 ± (2.74) | 3.49 ± (5.57) |
| BNP | 70.57 ± (94.95) | 194.92 ± (634.53) | 65.31 ± (133.36) | 201.75 ± (208.52) | 59.88 ± (93.5) |
| Procalcitonin | 1.7 ± (5.32) | 0.29 ± (0.84) | 0.71 ± (2.02) | 0.68 ± (1.58) | 1.09 ± (4.23) |
| Total calcium | 8.46 ± (0.64) | 8.8 ± (0.53) | 8.87 ± (0.72) | 8.6 ± (0.68) | 8.68 ± (0.66) |
| Sodium | 136.4 ± (4.69) | 137.04 ± (3.92) | 137.45 ± (4.02) | 137.61 ± (4.09) | 138.1 ± (3.4) |
| Chloride | 99.85 ± (5.01) | 100.72 ± (4.56) | 100.46 ± (4.54) | 101.68 ± (5.17) | 102.22 ± (3.87) |
| Potassium | 4.18 ± (0.54) | 4.24 ± (0.45) | 4.28 ± (0.46) | 4.27 ± (0.61) | 4.28 ± (0.45) |
| Phosphorus | 3.26 ± (0.84) | 3.36 ± (0.64) | 3.48 ± (0.69) | 3.37 ± (1.02) | 3.46 ± (0.79) |
| Hb | 11.64 ± (1.87) | 12.68 ± (2.02) | 12.15 ± (1.75) | 11.32 ± (2.17) | 12.33 ± (2.3) |
| RDW | 13.71 ± (1.57) | 13.47 ± (1.86) | 13.65 ± (1.84) | 15.38 ± (1.92) | 14.1 ± (2.15) |
| WBC | 12.89 ± (6.4) | 7.19 ± (2.29) | 8.86 ± (5.15) | 6.7 ± (2.66) | 10.45 ± (20.24) |
| MCHC | 33.05 ± (1.16) | 33.14 ± (1.26) | 32.86 ± (1.14) | 32.58 ± (1.13) | 32.96 ± (1.45) |
| MCV | 91.22 ± (5.81) | 90.32 ± (6.47) | 90.6 ± (5.31) | 93.35 ± (6.82) | 90.83 ± (6.7) |
| PDW | 10.4 ± (1.66) | 10.01 ± (2.16) | 10.38 ± (1.66) | 10.95 ± (2.67) | 10.34 ± (1.64) |
| Hct | 35.17 ± (5.41) | 38.15 ± (5.41) | 36.95 ± (5.06) | 34.7 ± (6.19) | 37.49 ± (6.76) |
| E-ANC | 10086.52 ± (6001.89) | 4819.34 ± (1999.56) | 6342.05 ± (4702.18) | 4474.5 ± (2465.85) | 6003.76 ± (4072.46) |
| MCH | 30.15 ± (2.31) | 29.93 ± (2.46) | 29.78 ± (2.11) | 30.43 ± (2.76) | 29.96 ± (2.7) |
| RBC | 3.87 ± (0.62) | 4.24 ± (0.65) | 4.09 ± (0.61) | 3.74 ± (0.76) | 4.15 ± (0.8) |
| MPV | 9.59 ± (0.81) | 9.23 ± (1.51) | 9.56 ± (1.14) | 9.89 ± (1.84) | 9.53 ± (1.15) |
| Platelet | 339.4 ± (136.28) | 309.02 ± (108.06) | 312.38 ± (114.78) | 208.67 ± (100.2) | 283.91 ± (115.53) |

ADA: Adenosine De-Aminase; ALT(SGPT): Alanine Transaminase; ANC: Absolute Neutrophil Count; AST(SGOT): Aspartate Transaminase; BNP: Brain Natriuretic Peptide; BUN: Blood Urea Nitrogen; CRP: C-Reactive Protein; Hb: Hemoglobin; LD: Lactate Dehydrogenase; MCH: Mean Corpuscular Hemoglobin; MCHC: Mean Cell Hemoglobin Concentration; MCV: Mean Corpuscular Volume; MPV: Mean Platelet Volume; PDW: Platelet Distribution Width; RBC: Red Blood Cell; RDW: Red cell Distribution Width; r-GT: Gamma-Glutamyl Transferase; WBC: White Blood Cell

# **Supplement Table 3. Confusion matrix of the validation set and extra-validation set predicted by the contrastive-loss model**

| **Validation set** | | | | | | |
| --- | --- | --- | --- | --- | --- | --- |
|  | | **Predicted** | | | | |
|  |  | Bacterial | Tuberculosis | Malignancy | Volume overload | Other |
| Targeted | Bacterial | 49 | 12 | 4 | 3 | 0 |
|  | Tuberculosis | 1 | 30 | 2 | 2 | 0 |
|  | Malignancy | 15 | 11 | 221 | 3 | 0 |
|  | Volume overload | 1 | 0 | 2 | 29 | 0 |
|  | Other | 5 | 5 | 8 | 1 | 0 |
| **Extra-validation set** | | | | | | |
|  | | **Predicted** | | | | |
|  |  | Bacterial | Tuberculosis | Malignancy | Volume overload | Other |
| Targeted | Bacterial | 110 | 8 | 41 | 2 | 0 |
|  | Tuberculosis | 8 | 89 | 9 | 0 | 0 |
|  | Malignancy | 8 | 13 | 246 | 7 | 0 |
|  | Volume overload | 5 | 0 | 18 | 19 | 0 |
|  | Other | 18 | 14 | 82 | 4 | 0 |

# **Supplement Table 4. The odds ratio of each laboratory result by multinomial logistic regression**

|  | **Class** | **Bacterial** | **Malignancy** | **Others** | **Tuberculosis** |
| --- | --- | --- | --- | --- | --- |
| Pleural  fluid | ADA | 1.57 (1.13–2.17) | 1.26 (0.91–1.74) | 1.4 (1.01–1.96) | 2.15 (1.53–3.02) |
|  | LD | 1.07 (1.03–1.11) | 1.08 (1.04–1.12) | 1.07 (1.03–1.11) | 1.07 (1.03–1.11) |
|  | Albumin | 2.12 (0.68–6.58) | 4.75 (1.68–13.4) | 1.93 (0.59–6.28) | 7.28(1.76–30.07) |
|  | Histiocyte | 0.72 (0.60–0.86) | 0.80 (0.68–0.94) | 0.81 (0.66–0.99) | 1.00 (0.76–1.31) |
|  | Neutrophil | 0.99 (0.85–1.14) | 0.89 (0.77–1.03) | 1.07 (0.91–1.26) | 1.28 (1.04–1.58) |
|  | Eosinophil | 0.68 (0.32–1.46) | 1.04 (0.58–1.85) | 2.04 (1.10–3.77) | 3.67 (1.61–8.39) |
|  | Lymphocyte | 0.90 (0.78–1.05) | 0.98 (0.85–1.12) | 1.04 (0.88–1.22) | 1.51 (1.21–1.87) |
|  | Mesothelial | 1.20 (0.39–3.63) | 0.72 (0.26–2.02) | 1.04 (0.3–3.67) | 0.66 (0.12–3.60) |
| Blood | AST | 0.99 (0.95–1.02) | 0.96 (0.93–0.99) | 0.99 (0.95–1.03) | 0.96 (0.92–1.01) |
|  | ALP | 1.06 (0.98–1.14) | 1.13 (1.05–1.21) | 1.08 (1.00–1.17) | 1.03 (0.93–1.14) |
|  | Albumin | 0.14 (0.03–0.65) | 0.06 (0.01–0.26) | 0.07 (0.01–0.38) | 0.19 (0.03–1.34) |
|  | Total protein | 0.54 (0.22–1.31) | 0.45 (0.20–1.03) | 0.71 (0.27–1.85) | 0.39 (0.13–1.17) |
|  | LD | 0.95 (0.92–0.99) | 1.00 (0.96–1.03) | 0.97 (0.92–1.01) | 0.96 (0.92–1.01) |
|  | CRP | 2.40 (1.23–4.68) | 0.98 (0.50–1.92) | 1.28 (0.62–2.64) | 2.30 (1.04–5.10) |
|  | BNP | 0.98 (0.95–1.01) | 0.93 (0.91–0.96) | 0.95 (0.92–0.98) | 0.98 (0.94–1.02) |
|  | Calcium | 1.42 (0.48–4.18) | 3.31 (1.21–9.03) | 1.42 (0.44–4.53) | 1.78 (0.47–6.73) |
|  | Hemoglobin | 0.37 (0.22–0.61) | 0.52 (0.33–0.82) | 0.42 (0.23–0.74) | 0.83 (0.41–1.67) |
|  | RDW | 0.07 (0.02–0.33) | 0.05 (0.01–0.20) | 0.06 (0.01–0.32) | 0.43 (0.05–3.53) |
|  | WBC | 0.89 (0.75–1.06) | 0.94 (0.80–1.10) | 0.87 (0.72–1.05) | 0.82 (0.64–1.05) |
|  | MCV | 1.96 (1.29–2.98) | 1.17 (0.81–1.70) | 1.34 (0.85–2.11) | 1.11 (0.63–1.96) |
|  | RBC | 25.36 (6.76–95.05) | 12.04 (3.77–38.43) | 43.60 (9.41–201.98) | 2.85 (0.45–18.20) |
|  | Platelet | 0.98 (0.95–1.01) | 0.98 (0.96–1.01) | 0.96 (0.93–0.99) | 1.01 (0.97–1.05) |

Reference value: volume overload. The model-dependent variables were selected by backward elimination.

ADA: Adenosine De-Aminase; ALT(SGPT): Alanine Transaminase; AST(SGOT): ASpartate Transaminase; BNP: Brain Natriuretic Peptide; CRP: C-Reactive Protein; LD: Lactate Dehydrogenase; MCV: Mean Corpuscular Volume; RBC: Red Blood Cell; RDW: Red cell Distribution Width; WBC: White Blood Cell

# **Supplement Table 5. List of extracted chemical categories for blood and pleural effusion.**

| Panel of the test | Type of the laboratory test |
| --- | --- |
| Pleural cell count | Histiocyte [Cytospin, Wright’s stain]  Neutrophil [Cytospin, Wright’s stain]  RBC [Cytospin, Wright’s stain]  Eosinophil [Cytospin, Wright’s stain]  Nucleated cells [Cytospin, Wright’s stain]  Lymphocyte [Cytospin, Wright’s stain]  Mesothelial cell [Cytospin, Wright’s stain]  Atypical lymphocyte [Cytospin, Wright’s stain]  Malignant cell [Cytospin,Wright’s stain] “,  Atypical cell [Cytospin, Wright’s stain]  Basophil [Cytospin, Wright’s stain]  Other cells [Cytospin, Wright’s stain]  Leukemic blast [Cytospin, Wright’s stain].” |
| Pleural fluid | ‘ADA’, ‘LD’, ‘albumin’, ‘Total protein’, ‘Glucose’, ‘pH ‘, ‘Total amylase’, ‘Lipase’ |
| CBC | ‘Hb’, ‘RDW’, ‘WBC’, ‘MCHC’, ‘MCV’, ‘PDW’,  ‘Hct’, ‘ANC’, ‘MCH’, ‘RBC’, ‘MPV’, ‘Platelet’ |
| Chemistry | ‘Creatinine’, ‘BUN’, ‘AST(SGOT)’, ‘ALT(SGPT)’, ‘Alkaline phosphatase’, ‘r-GT’, ‘Total bilirubin’, ‘Direct bilirubin’, ‘Glucose’, ‘Total protein’, ‘Albumin’, ‘Amylase’, ‘Lipase’, ‘BNP’, ‘CRP’, ‘Procalcitonin’, ‘Sodium’, ‘Chloride’, ‘Potassium’, ‘Phosphorus’, ‘Ionized calcium’, ‘Total calcium’, ‘LD’, ‘D-dimer’, ‘ANA titer’, ‘ANA 1:40’, ‘Rheumatoid factor’ |

ADA: Adenosine De-Aminase; ALT(SGPT): Alanine Transaminase; ANA: Anti-Nuclear Antibody; ANC: Absolute Neutrophil Count; AST(SGOT): ASpartate Transaminase; BNP: Brain Natriuretic Peptide; BUN: Blood Urea Nitrogen; CRP: C-Reactive Protein; Hb: Hemoglobin; Hct: Hematocrit; LD: Lactate Dehydrogenase; MCH: Mean Corpuscular Hemoglobin; MCHC: Mean Cell Hemoglobin Concentration; MCV: Mean Corpuscular Volume; MPV: Mean Platelet Volume; PDW: Platelet Distribution Width; RBC: Red Blood Cell; RDW: Red cell Distribution Width; r-GT: Gamma-GlutamylTransferase; WBC: White Blood Cell

# **Supplement Table 6. Interobserver agreement and Cohen’s Kappa in the extra-validation dataset.**

|  |  | Reviewer 1 | | | | |
| --- | --- | --- | --- | --- | --- | --- |
|  |  | **Bacterial infection** | **Tuberculosis** | **Malignancy** | **Volume overload** | **Other** |
| Reviewer 2 | **Bacterial infection** | 144 | 3 | 3 | 1 | 9 |
|  | **Tuberculosis** | 1 | 97 | 0 | 0 | 0 |
|  | **Malignancy** | 4 | 0 | 223 | 0 | 5 |
|  | **Volume overload** | 0 | 1 | 0 | 34 | 1 |
|  | **Other** | 14 | 15 | 56 | 3 | 87 |

|  |  | Reviewer 1 | | | | |
| --- | --- | --- | --- | --- | --- | --- |
|  |  | **Bacterial infection** | **Tuberculosis** | **Malignancy** | **Volume overload** | **Others** |
| Reviewer 2 | **Bacterial infection** | 0.859 |  |  |  |  |
|  | **Tuberculosis** |  | 0.890 |  |  |  |
|  | **Malignancy** |  |  | 0.792 |  |  |
|  | **Volume overload** |  |  |  | 0.914 |  |
|  | **Others** |  |  |  |  | 0.544 |

Cohen’s Kappa value of interobserver agreement in the extra-validation set was 0.780. Cohen’s Kappa value shows the interobserver agreement on each etiology.

# **Supplement Table 7. List of 46 categories for model development.**

| Panel of the test | Type of the laboratory test |
| --- | --- |
| Pleural chemistry | ‘ADA’, ‘LD’, ‘Albumin’, ‘pH ‘, ‘Total protein’, ‘Glucose’, ‘Amylase’ |
| Pleural cytology | ‘Histiocyte,’ ‘Neutrophil,’ ‘RBC,’ ‘Nucleated cells,’ ‘Lymphocyte’  ‘Eosinophil,’ ‘Mesothelial cell’ |
| Blood chemistry | ‘Total calcium’, ‘Phosphorus’, ‘Creatinine’, ‘BUN’, ‘Glucose’, ‘Total protein’, ‘Albumin’, ‘ALT(SGPT)’, ‘AST(SGOT)’, ‘Total bilirubin’, ‘r-GT’, ‘Alkaline phosphatase’, ‘Sodium’, ‘Chloride’, ‘Potassium’, ‘D-dimer’, ‘BNP’, ‘CRP’, ‘procalcitonin’, ‘LD’ |
| CBC | ‘WBC’, ‘E-ANC’, ‘RBC’, ‘Hemoglobin’, ‘RDW’, ‘MCHC’, ‘MCV’, ‘PDW’, ‘Hct’, ‘MCH’, ‘MPV’, ‘Platelet’, |

The above list was selected from those below 40% of the omission rate.

ADA: Adenosine De-Aminase; ALT(SGPT): Alanine Transaminase; ANC: Absolute Neutrophil Count; AST(SGOT): ASpartate Transaminase; BNP: Brain Natriuretic Peptide; BUN: Blood Urea Nitrogen; CRP: C-Reactive Protein; Hct: Hematocrit; LD: Lactate Dehydrogenase; MCH: Mean Corpuscular Hemoglobin; MCHC: Mean Cell Hemoglobin Concentration; MCV: Mean Corpuscular Volume; MPV: Mean Platelet Volume; PDW: Platelet Distribution Width; RBC: Red Blood Cell; RDW: Red cell Distribution Width; r-GT: Gamma-GlutamylTransferase; WBC: White Blood Cell

# **References**

1. El-Habil, A. M. An application on multinomial logistic regression model. *Pakistan J. Stat. Oper. Res.* **8**, 271–291 (2012).

2. Breiman, L. Random_Forest. *Mach. Learn.* **45**, 5–32 (2001).

3. Murtagh, F. Multilayer perceptrons for classification and regression. *Neurocomputing* **2**, 183–197 (1991).

4. Nwankpa, C. E., Ijomah, W., Gachagan, A. & Marshall, S. Activation functions: Comparison of trends in practice and research for deep learning. *arXiv* 1–20 (2018).

5. Döring, M. Performance Measures for Multi-Class Problems - Data Science Blog: Understand. Implement. Succed.

6. Joseph, S. Australian Literary Journalism and “Missing Voices”: How Helen Garner finally resolves this recurring ethical tension. *Journal. Pract.* **10**, 730–743 (2016).

7. Khosla, P. *et al.* Supervised contrastive learning. *arXiv* 1–23 (2020).

8. Glorot, X. & Bengio, Y. Understanding the difficulty of training deep feedforward neural networks. *J. Mach. Learn. Res.* **9**, 249–256 (2010).

9. Paszke, A. *et al.* PyTorch: An imperative style, high-performance deep learning library. *arXiv* (2019).

10. Kingma, D. P. & Ba, J. L. Adam: A Method for Stochastic Optimization. 1–15 (2015).
